# Proximity Staining Using Enzymatic Protein Tagging in Diplomonads

**DOI:** 10.1128/mSphereDirect.00153-19

**Published:** 2019-03-20

**Authors:** Ásgeir Ástvaldsson, Kjell Hultenby, Staffan G. Svärd, Jon Jerlström-Hultqvist

**Affiliations:** a Department of Cell and Molecular Biology, BMC, Uppsala University, Uppsala, Sweden; b Department of Laboratory Medicine, Karolinska Institutet, Stockholm, Sweden; University at Buffalo; Institute of Parasitology, University of Zürich; University of California, Davis

**Keywords:** APEX, DAB, *Giardia*, proximity labeling, *Spironucleus salmonicida*

## Abstract

The diplomonads are a group of understudied eukaryotic flagellates whose most prominent member is the human pathogen Giardia intestinalis. Methods commonly used in other eukaryotic model systems often require special optimization in diplomonads due to the highly derived character of their cell biology. We have optimized a proximity labeling protocol using pea ascorbate peroxidase (APEX) as a reporter for transmission electron microscopy (TEM) to enable the study of ultrastructural cellular details in diplomonads. Currently available TEM-compatible tags require light-induced activation (1, 2) or are inactive in many cellular compartments (3), while ascorbate peroxidase has not been shown to have those limitations. Here, we have optimized the *in vivo* activities of two versions of pea ascorbate peroxidase (APX^W41F^ and APEX) using the diplomonad fish parasite Spironucleus salmonicida, a relative of G. intestinalis. We exploited the well-known peroxidase substrates, Amplex UltraRed and 3,3′-diaminobenzidine (DAB), to validate the activity of the two tags and argue that APEX is the most stable version to use in Spironucleus salmonicida. Next, we fused APEX to proteins with established localization to evaluate the activity of APEX in different cellular compartments of the diplomonad cell and used Amplex UltraRed as well as antibodies along with superresolution microscopy to confirm the protein-APEX localization. The ultrastructural details of protein-APEX fusions were determined by TEM, and we observed marker activity in all cellular compartments tested when using the DAB substrate. Finally, we show that the optimized conditions established for S. salmonicida can be used in the related diplomonad G. intestinalis.

**IMPORTANCE** The function of many proteins is intrinsically related to their cellular location. Novel methods for ascertainment of the ultrastructural location of proteins have been introduced in recent years, but their implementation in protists has so far not been readily realized. Here, we present an optimized proximity labeling protocol using the APEX system in the salmon pathogen Spironucleus salmonicida. This protocol was also applicable to the human pathogen Giardia intestinalis. Both organisms required extraneous addition of hemin to the growth medium to enable detectable peroxidase activity. Further, we saw no inherent limitation in labeling efficiency coupled to the cellular compartment, as evident with some other proximity labeling systems. We anticipate that the APEX proximity labeling system might offer a great resource to establish the ultrastructural localization of proteins across genetically tractable protists but might require organism-specific labeling conditions.

## Abstract (precis)

The function of many proteins is intrinsically related to their cellular location. Novel methods for ascertainment of the ultrastructural location of proteins have been introduced in recent years, but their implementation in protists has so far not been readily realized. Here, we present an optimized proximity labeling protocol using the APEX system in the salmon pathogen Spironucleus salmonicida. This protocol was also applicable to the human pathogen Giardia intestinalis. Both organisms required extraneous addition of hemin to the growth medium to enable detectable peroxidase activity. Further, we saw no inherent limitation in labeling efficiency coupled to the cellular compartment, as evident with some other proximity labeling systems. We anticipate that the APEX proximity labeling system might offer a great resource to establish the ultrastructural localization of proteins across genetically tractable protists but might require organism-specific labeling conditions.

## INTRODUCTION

The diplomonads are a diverse group of unicellular flagellated microorganisms that are binucleated and lack some typical eukaryotic features, e.g., Golgi and mitochondria capable of oxidative phosphorylation, although mitochondrial remnant organelles (MROs) have been identified, e.g., mitosomes in Giardia spp. and hydrogenosomes in Spironucleus salmonicida (4–6). The highly derived cell biology of the binucleated and tetraploid diplomonad cells as well as high proportion of hypothetical proteins uncovered in genome sequencing efforts present special experimental challenges. Furthermore, even related lineages, like Giardia and Spironucleus spp., contain thousands of lineage-specific genes whose functions cannot be easily inferred by homology (7). There has been steady progress in developing diplomonad genetic tools, including successful demonstrations of genetically encoded ultrastructural labeling in Giardia intestinalis (8) and in S. salmonicida (9), but the methods have not been evaluated and optimized in a systematic way in any diplomonad.

Horseradish peroxidase (HRP) has been used as a genetically encoded fusion partner, but a number of shortcomings, such as sensitivity to the redox state of a cell compartment and the necessity to coordinate Ca^2+^ ions, have limited its use (10). HRP is also sensitive to inactivation by aldehyde-based fixatives and does not exhibit robust activity after fixation optimal for the preservation of cell ultrastructure. The class 2 peroxidases, including ascorbate peroxidase (APX) from legumes, lack disulfide bonds, have no need to coordinate Ca^2+^ ions, and are active even after harsh chemical fixation (11). Recently, the Ting lab developed the pea ascorbate peroxidase (APEX), a mutated version of APX, which is monomeric and active in all cellular compartments tested (11). APEX is active with a range of different substrates, including 10-acetyl-3,7-dihydroxyphenoxazine (Amplex UltraRed) and 3,3′-diaminobenzidine (DAB). When transfected cells expressing the peroxidase are immersed in a solution of Amplex UltraRed and hydrogen peroxide (H_2_O_2_), the peroxidase oxidizes the substrates to generate a precipitate called resorufin at the location of the tagged protein, which can be imaged by fluorescence microscopy (11). Using DAB is analogous to the reaction with Amplex UltraRed, but here, peroxidase catalyzes the oxidation of the DAB substrate in the presence of H_2_O_2_, generating a dark brown alcohol-insoluble precipitate at the location of the protein of interest (11). The precipitate is visible by light microscopy, and the osmiophilic nature of the precipitate enables enhanced contrast by transmission electron microscopy (TEM) (see Fig. S1 in the supplemental material) (11).

10.1128/mSphereDirect.00153-19.1 FIG S1 The APEX reaction. A schematic illustration of the H_2_O_2_-dependent oxidation of Amplex UltraRed and DAB catalyzed by APEX. Download FIG S1, PDF file, 0.1 MB. Copyright © 2019 Ástvaldsson et al. 2019 Ástvaldsson et al. This content is distributed under the terms of the Creative Commons Attribution 4.0 International license.

Here, we describe an optimized protein proximity labeling protocol in the diplomonad S. salmonicida using the pea version of APEX as a fusion partner (11), thereby expanding the method toolkit already established in this organism (12). We also demonstrate that the optimized protocol is applicable to the diplomonad model organism G. intestinalis, the most studied diplomonad parasite. The comparative ease of use of the APEX system will make high-resolution ultrastructural labeling more tractable and not exclusively reliant on antibody staining. It will also permit high-resolution comparative ultrastructure localization studies of shared diplomonad traits.

## RESULTS

### Initial optimization of ascorbate peroxidase activity.

We generated transfected strains carrying episomal vectors that expressed protein fusions to two mutated variants of pea ascorbate peroxidase, ^W41F^APX and APEX, to evaluate and optimize their activities in S. salmonicida. We tagged a well-characterized structural protein, annexin 5 (SS50377_10477), which has a very distinct localization in the anterior end of the cell, resembling a hat-like structure of unknown function (9). Initial experiments showed no clear evidence of ^W41F^APX or APEX-derived peroxidase activity in the transfected cells when grown in the standard liver digest-yeast extract-iron (LYI) growth medium, even though we were able to demonstrate the presence of the expected full-length protein and its correct subcellular localization (see Fig. S2A). We reasoned that a lack of peroxidase activity might be connected to low bioavailability and incorporation of the heme cofactor since diplomonad cells do not synthesize their own heme (13). Interestingly, Giardia spp. are still able to scavenge sufficient heme to accommodate at least five heme-incorporating proteins (13). Homologues to those very same proteins are also present in the genome of S. salmonicida, indicating that it may also be able to scavenge heme from the extracellular milieu. To boost APEX activity, we grew our transgenic cell lines in LYI medium supplemented with 200 µM hemin. Under these growth conditions, we noticed pronounced *in vivo* APEX activity, validating our suspicions of low bioavailability of hemin as the reason for a lack of APEX activity.

10.1128/mSphereDirect.00153-19.2 FIG S2 Western blot analyses of APEX-V5*-*tagged proteins in transfectant strains of *S. salmonicida* (A) and *G. intestinalis* (B). Transfectant and wild-type (WT) cells were grown in LYI medium (A) and TYDK medium (B). Cultures were collected and boiled for 10 min in Laemmli buffer containing 100 mM DTT. The V5 tag was detected using the primary monoclonal mouse anti-V5 antibody and polyclonal rabbit anti-mouse HRP*-*conjugated antibody as secondary. Theoretical molecular masses (from left to right) are Anx5, 64.9 kDa; IscU, 45.25 kDa; SHMT, 75.23 kDa; IscS, 72.92 kDa; PFOR5, 146.17 kDa; 10316, 152.88 kDa; His3var, 44.85 kDa; BiP, 100.9 kDa; acid phosphatase, 70.18 kDa; NADH, 72.10 kDa; fibrillarin, 64.67 kDa; and IFT46, 60.63 kDa (A), and SALP-1, 59.07 kDa; alpha-14 giardin, 67.83 kDa; alpha-19 giardin, 77.30 kDa; IscU, 52.51 kDa; IscS, 47.69 kDa; HisH3B, 17.77 kDa; BiP, 74.36 kDa; and acid phosphatase, 75.20 kDa (B). Download FIG S2, PDF file, 0.5 MB. Copyright © 2019 Ástvaldsson et al. 2019 Ástvaldsson et al. This content is distributed under the terms of the Creative Commons Attribution 4.0 International license.

### Optimization of hemin and hydrogen peroxide concentrations.

Excessive hemin concentrations are known to be detrimental to cells due to the production of reactive oxygen species or membrane peroxidation (14, 15). In line with these observations, we noticed poor growth and changes to cell morphology at 200 µM hemin supplementation. We sought to minimize the concentration of added hemin to establish a balance between cell viability and APEX activity. The transfectants were grown in five concentrations of hemin (0 to 200 µM), and the optimal hemin concentration was then estimated using the readout from both DAB and Amplex UltraRed substrate staining (see Fig. S3A). DAB and Amplex UltraRed were reacted with 10 mM and 8.5 mM H_2_O_2_ for 15 and 30 min, respectively. At the lowest hemin concentration, 40 µM, we observed healthy cells with the typical pyriform appearance, but the DAB signal was reduced compared to that with higher concentrations. The optimal hemin concentration was estimated to be between 80 and 120 µM since at these concentrations, the cells displayed pyriform morphology, could be maintained by weekly passage using an inoculum similar to that in in hemin-free medium, and showed easily detectable peroxidase activity with distinct localization. Using Amplex UltraRed as the substrate yielded similar results, but at higher hemin supplementation concentrations, the fluorescent signal became more diffusely localized (see Fig. S3B).

10.1128/mSphereDirect.00153-19.3 FIG S3 Hemin titrations. *S. salmonicida* transfectants expressing either Anx5-APEX or Anx5-APX were grown in LYI medium containing different amounts of hemin. Cells were spun down and washed with HBSS-G before they were spotted on a poly-lysine-coated microscopy slide. (A) HBSS-G was removed, and cells was fixed for 30 to 60 min with 2% glutaraldehyde in 100 mM cacodylate buffer with 2 mM CaCl_2_. The cells were reacted with 0.5 mg/ml DAB substrate and 10 mM H_2_O_2_ for 15 min and extensively washed with cacodylate buffer and PBS. The slide was mounted with VectaShield. Cells with white deposits in the anterior of the cells are positive for Anx5-APEX or Axn5-APX staining. (B) The cells are fixed with 2% paraformaldehyde in PBS before being reacted with 50 µM Amplex UltraRed and 8.5 mM H_2_O_2_ for 30 min. Cells were washed extensively with PBS and mounted with VectaShield with DAPI. Scale bars = 10 µm. Cells with red deposits in the anterior of the cells are positive for Anx5-APEX or Axn5-APX staining. Download FIG S3, PDF file, 1.9 MB. Copyright © 2019 Ástvaldsson et al. 2019 Ástvaldsson et al. This content is distributed under the terms of the Creative Commons Attribution 4.0 International license.

**FIG 1:**
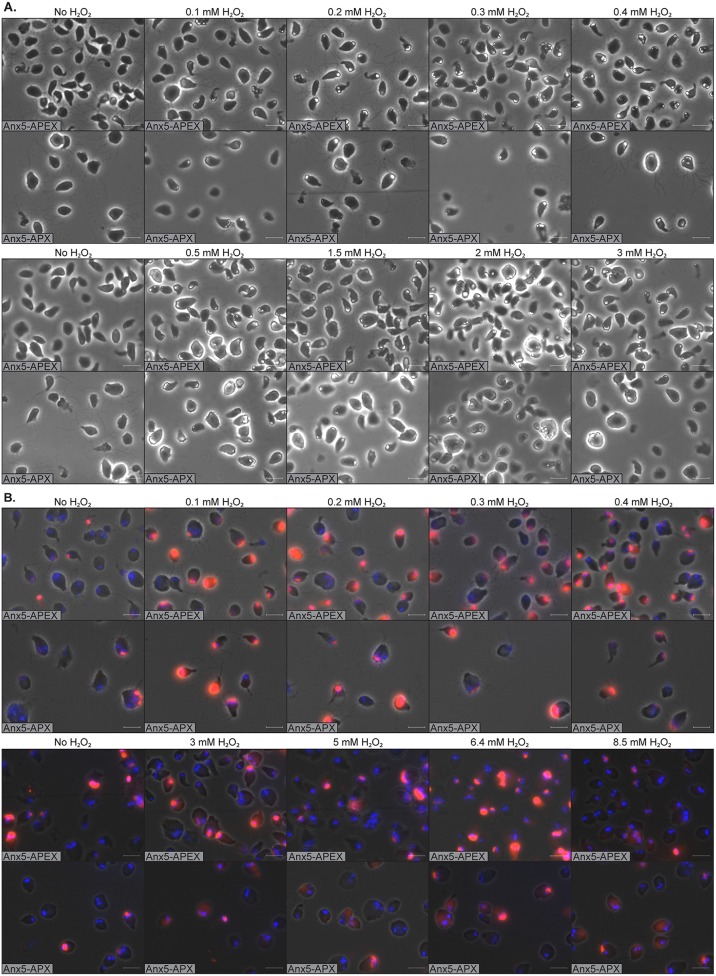
H_2_O_2_ titrations. *S. salmonicida* transfectants expressing Anx5-APEX or Anx5-APX were grown in LYI medium supplemented with 100 µM hemin. Transfectants were washed with HBSS-G and spotted on a poly-lysine*-*coated microscopy slide. (A) Cells were fixed with 2% glutaraldehyde in 100 mM cacodylate buffer with 2 mM CaCl_2_ before being reacted with 0.5 mg/ml DAB and 0 to 3 mM H_2_O_2_ for 15 min. Cells were washed with cacodylate buffer and PBS, mounted with VectaShield, and viewed in a phase-contrast microscope. Cells with white deposits in the anterior of the cells are positive for Anx5-APEX or Axn5-APX staining. (B) Cells were fixed with 2% paraformaldehyde in PBS and reacted with 50 µM Amplex UltraRed and 0 to 8.5 mM H_2_O_2_ for 30 min. The cells were washed extensively, mounted with VectaShield with DAPI, and imaged in a fluorescence microscope. Cells with red deposits in the anterior of the cells are positive for Anx5-APEX or Axn5-APX staining. Scale bars = 10 µm.

To establish further the optimal conditions for the peroxidase activity while maintaining cell survival and integrity, we used the DAB substrate and titrated the H_2_O_2_ concentration in a range of 0 to 3 mM while keeping the hemin concentration constant at 100 µM. With no addition of H_2_O_2_, the signal is effectively quenched, although we noticed some residual signal that might represent endogenous peroxidase activity. The signal is strongly stimulated by a low concentration of H_2_O_2_, and it does not significantly change with concentrations of 200 µM or above. High H_2_O_2_ concentrations negatively affected the cells, and the signal became more delocalized, even if the cells were fixed with glutaraldehyde or paraformaldehyde prior to labeling with the substrates (Fig. 1A). The above-mentioned observations were also found to hold true for the Amplex UltraRed substrate (Fig. 1B). A comparison between APEX and ^W41F^APX during the hemin and H_2_O_2_ titration experiments showed that the activity of APEX is much higher than that of ^W41F^APX at low hemin and H_2_O_2_ concentrations. The signal generated by ^W41F^APX is also more prone to be delocalized, while the APEX signal is more distinct and localized to the expected cellular structures (Fig. 1 and S3).

### APEX is functional in different cell compartments of the diplomonad cell.

We evaluated the broad functionally of APEX in diplomonads by constructing a collection of C-terminal protein fusions (13 proteins in S. salmonicida and 11 in G. intestinalis) (Table 1 and Fig. S4). The expression was provided by the native promoter of each gene from the context of an episomal plasmid vector. The localizations of most of these proteins were known *a priori* to allow us to inspect the activity level of APEX in different cell compartments. However, we also included two proteins with uncertain or previously unknown localization to demonstrate the utility of APEX in the characterization of novel proteins (Table 1). We appended a C-terminal V5 epitope tag to all the constructs to be able to independently confirm the localization and expression of the APEX fusion using immunofluorescence and Western blotting. APEX activity was then investigated using Amplex UltraRed and DAB, as described previously. We were unable to generate viable transfectant lines for histone H3 (SS50377_17319) and histone H3B (SS50377_17654) in S. salmonicida and fibrillarin (GL50803_97219), Cen H3 (GL50803_20037), and histone H3 (GL50803_135231) in G. intestinalis. We were able to confirm the expression of most protein-APEX-V5 fusions in S. salmonicida transfectants by Western blotting, and the observed molecular weights were in line with the predicted *in silico* values. The exception was the nuclear protein histone H3 variant (SS50377_10544), where no protein expression was observed (see Fig. S2A). In the transgenic G. intestinalis strains, we only detected the expected protein species expressed for three proteins, striated fiber assemblin-like protein 1 (SALP-1; GL50803_4410), alpha-14 giardin (GL50803_15097), and IscU (GL50803_15097). Additional N-terminal degradation products were detected for binding immunoglobulin protein (BiP; SS50377_11120) in S. salmonicida and alpha-14 giardin in G. intestinalis (see Fig. S2B).

**TABLE 1:** Full list of *S. salmonicida* and *G. intestinalis* proteins used in this study with GiardiaDB accession numbers, sizes, and localizations

Hypothetical protein	Organism	GiardiaDB accession no.	Size (bp)	Localization (reference)
10316	*S. salmonicida*	SS50377_10316	3,242	Two foci in anterior end of the cell (4)
Acid phosphatase	*S. salmonicida*	SS50377_10140	1,082	Endoplasmic reticulum-predicted localization
Annexin 5	*S. salmonicida*	SS50377_10477	932	Unknown structure in anterior of the nuclei (12)
BiP	*S. salmonicida*	SS50377_11120	1,925	Endoplasmic reticulum (12)
Fibrillarin	*S. salmonicida*	SS50377_13348	971	Nuclei (12)
Histone H3	*S. salmonicida*	SS50377_17319	431	Nuclei (predicted localization)
NADH oxidase	*S. salmonicida*	SS50377_12178	1,133	Perinuclear region around the nuclei (4)
Histone H3B	*S. salmonicida*	SS50377_17654	440	Nuclei (predicted localization)
Histone H3var	*S. salmonicida*	SS50377_10544	401	Nuclei (predicted localization)
IFT46	*S. salmonicida*	SS50377_16623	818	Flagella (12)
IscS	*S. salmonicida*	SS50377_17654	1,197	Hydrogenosomes (4)
IscU	*S. salmonicida*	SS50377_11862	445	Hydrogenosomes (4)
PFOR5	*S. salmonicida*	SS50377_10765	3,140	Hydrogenosomes (4)
SHMT	*S. salmonicida*	SS50377_17865	1,241	Hydrogenosomes (4)
Acid phosphatase	*G. intestinalis*	GL50803_7556	1,202	Acid phosphatase activity in peripheral vesicles (29)
Alpha-14 giardin	*G. intestinalis*	GL50803_15097	1,010	Local slubs in flagella, and plasma membrane (18, 19)
Alpha-19 giardin	*G. intestinalis*	GL50803_4026	1,316	Ventral flagellum pair (30)
BiP	*G. intestinalis*	GL50803_17121	2,033	Endoplasmic reticulum (31)
Cen H3	*G. intestinalis*	GL50803_20037	470	Nuclei (32)
Fibrillarin	*G. intestinalis*	GL50803_97219	938	Nucleolus (33)
Histone H3	*G. intestinalis*	GL50803_135231	440	Nuclei (32)
Histone H3B	*G. intestinalis*	GL50803_3367	479	Nuclei (32)
IscS	*G. intestinalis*	GL50803_14519	1,301	Mitosomes (5)
IscU	*G. intestinalis*	GL50803_15196	638	Mitosomes (5)
SALP-1	*G. intestinalis*	GL50803_4410	764	Ventral disc (16, 17)

10.1128/mSphereDirect.00153-19.4 FIG S4 Schematic illustration of protein localizations from *S. salmonicida* (A) and *G. intestinalis* (B) relevant to the study. Text colors are as follows: black, successful transfection and expression; green, no viable transfectants obtained; and red, no expression detected. Download FIG S4, PDF file, 0.2 MB. Copyright © 2019 Ástvaldsson et al. 2019 Ástvaldsson et al. This content is distributed under the terms of the Creative Commons Attribution 4.0 International license.

**FIG 2:**
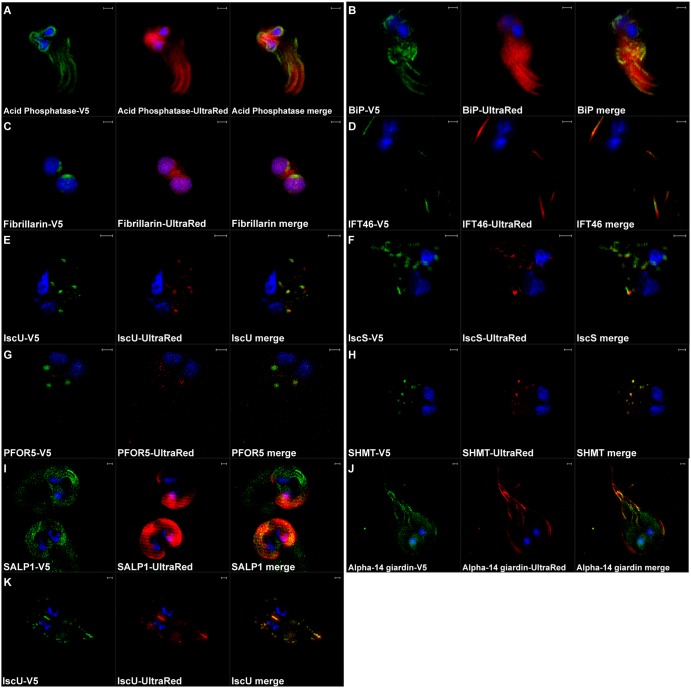
Superresolution microscopy (SIM) of *S. salmonicida* (A to H) and *G. intestinalis* (I and J) transfectants. Transfectants expressing APEX-V5 were grown in LYI medium (*S. salmonicida*) and TYDK medium (*G.* intestinalis) supplemented with 100 µM hemin. Cultures were fixed with 2% paraformaldehyde in PBS and treated with 50 µM Amplex UltraRed (Red) and 200 µM H_2_O_2_ for 30 min. V5 epitope (green) was detected using a primary monoclonal mouse anti-V5 antibody and a secondary polyclonal goat anti-mouse Alexa Fluor 488*-*conjugated antibody. The cells were stained with 2 µg/ml DAPI solution (blue) for 10 min and mounted with VectaShield. Imaging was done by a Zeiss LSM710 microscope with a SIM module. Scale bars = 1 µm.

Next, we used structured illumination superresolution microscopy (SIM) to determine the subcellular localization of the protein-APEX-V5 constructs. Amplex UltraRed was used to initially assay the activity of APEX in different cellular compartments, and we found that the resorufin and V5 signals were colocalized in all transfectant strains. In S. salmonicida, the V5 antibody stain is observed in a more specific pattern and can be resolved with more detail, while the resorufin signal is more diffuse (Fig. 2A to H). In G. intestinalis, the V5 antibody does not produce efficient labeling; instead, the resorufin signal is stronger. Again, the patterns of the antibody staining and the resorufin precipitates are closely matched (Fig. 2I to K). The expressed proteins in both S. salmonicida and G. intestinalis display localizations (see descriptions below) that correspond well with their previously published subcellular localization or the expected localization based on data from other eukaryotes (Table 1).

In determination of the localizations of two of the proteins in S. salmonicida, acid phosphatase and BiP, show labeling around the two nuclei stretching to the cell posterior along the recurrent flagellar axis. The area posterior to the nuclei showed an especially prominent stain. This labeling pattern is consistent with previous work that localized proteins to the endoplasmic reticulum (ER) in S. salmonicida (Fig. 2A and B) (12).

Fibrillarin was found to be localized to distinct subregions in both nuclei. The stain was often found in a diffuse pattern at the nuclear periphery in regions not overlapping the genomic DNA stain. The focus of the stain was often in the anterior part of the nucleus, but it was not uncommon to find parts of the signal at other nuclear locations as well. This localization is consistent with previous localization data in S. salmonicida (Fig. 2C) (12).

SIM localization of intraflagellar transport 46 (IFT46) protein revealed punctuate localization along the flagella and the basal bodies. There was a clear enrichment of signal in the distal ends of the flagella. Again, this localization is consistent with previous localization data in S. salmonicida (Fig. 2D) (12).

The hydrogenosome is a type of MRO previously described in S. salmonicida (4). We determined the localizations of four hydrogenosomal proteins (IscU, IscS, serine hydroxymethyltransferase [SHMT], and pyruvate ferredoxin oxidoreductase [PFOR5]) using APEX fusions. All four proteins showed a typical hydrogenosome staining pattern with tens of round to ovoid foci distributed in the cytosol (Fig. 2E to H) (4).

**FIG 3:**
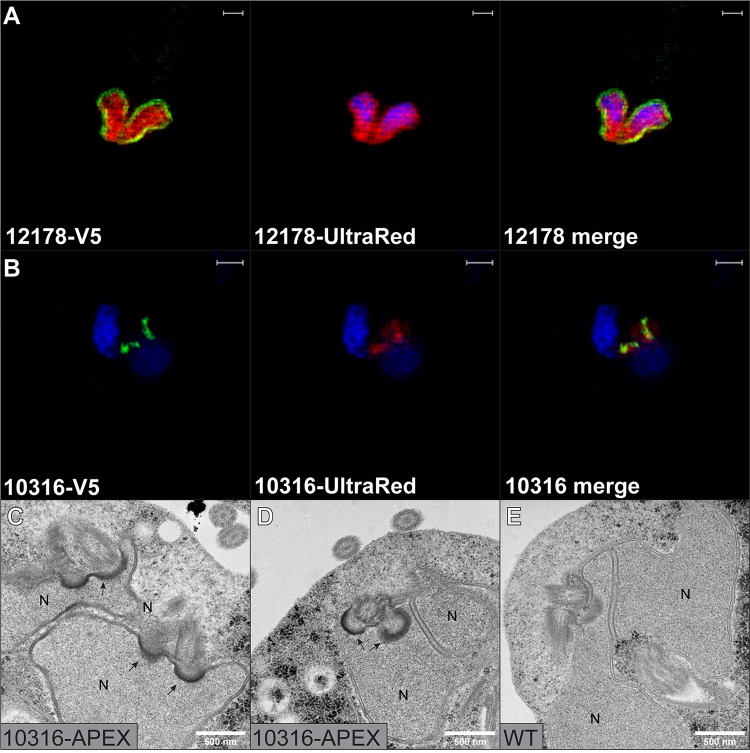
SIM and TEM images of two previously uncharacterized proteins. Transfectants expressing 10316-APEX-V5 and 12178-APEX-V5 were grown in LYI medium supplemented with 100 µM hemin. (A and B) SIM images. Transfectants were fixed with 2% paraformaldehyde in PBS and treated with 50 µM Amplex UltraRed (red) and 200 µM H_2_O_2_ for 30 min. V5 epitope (green) was detected using a primary monoclonal mouse anti-V5 antibody and a secondary polyclonal goat anti-mouse Alexa Fluor 488*-*conjugated antibody. The cells were stained with 2 µg/ml DAPI solution (blue) for 10 min and mounted with VectaShield. Imaging was done by a Zeiss LSM710 microscope with a SIM module. Scale bars = 1 µm. (C to E) TEM images. Transfectants were fixed with 2% glutaraldehyde in 100 mM cacodylate buffer with 2 mM CaCl_2_ and labeled with 0.5 mg/ml DAB and 300 µM H_2_O_2_ for 15 min. Samples are postfixed in 2% osmium tetroxide in 0.1 M phosphate buffer following dehydration in ethanol and acetone. Samples were embedded in LX-112 resin, and 50- to 60-nm sections were cut. Samples were viewed at 80 kV on a Hitachi HT 7700 lens and imaged with a Veleta camera. APEX-catalyzed DAB deposition appears as higher contrast areas at the site of the tagged protein. Some areas with labeling are indicated by arrows. Abbreviation: N nucleus. Scale bars are indicated in the images.

Last for S. salmonicida, we determined the localization of two previously uncharacterized proteins. SS50377_12178 is protein with homology to NADH:flavin oxidoreductases that has potentially been laterally transferred from prokaryotes. SS50377_12178 localized distinctly to the perinuclear region around both nuclei (Fig. 3A). In a previous localization effort attempting to identify novel hydrogenosomal proteins, SS50377_10316 was serendipitously localized to two foci of uncertain identity in the anterior end of the cell (4). SS50377_10316 is a hypothetical protein with no recognizable homologs outside diplomonads. Our SIM localization data confirmed the previous localization and were able to further resolve each focus as two elongate structures positioned in the anterior end of the cell in close proximity to the nuclei (Fig. 3B).

SALP-1 is a Giardia-specific ventral disc protein with homology to striated fiber assemblins (16, 17). SALP-1 tagged with APEX displayed a distinct localization to the ventral disc. V5 localization of SALP-1 suffered from poor signal, possibly due to difficulties in antibody accessibility (Fig. 2I).

In diplomonads, the annexins (in Giardia spp. also known as alpha-giardins) constitute an expanded family of proteins associated with the membrane and cytoskeleton. Alpha-14 giardin has previously been reported to localize to the flagella (18, 19). We obtained a clear localization of Amplex UltraRed to the flagella, whereas the V5 localization was only faint but primarily seen in the flagella (Fig. 2J).

Finally, we fused APEX to the mitosome protein IscU. Characteristically, SIM and Amplex UltraRed signals were seen to colocalize in a linear arrangement between the nuclei and at peripheral cellular sites typical of mitosome localization in Giardia spp. (Fig. 2K) (5).

In summary, we were able to obtain reliable protein localizations using the Amplex UltraRed substrate for all strains where an APEX fusion resulted in a demonstrable expression of a full-length protein. The resorufin signal generated by APEX was found to be less distinctly localized than the V5-tag derived signal when viewed using SIM. We were more successful in generating effective APEX fusions in S. salmonicida than in Giardia spp.

### Ultrastructural localization using APEX-catalyzed DAB deposition.

Finally, we investigated the ultrastructural localization of the tagged proteins in the successful APEX transgenic strains. The transfectants were grown according to the optimized protocol, being fixed in glutaraldehyde in cacodylate buffer before the DAB substrate is added to the samples. Directly after the DAB treatment, the cells were viewed with a transmitted light microscope to validate the labeling efficiency before TEM sample preparation (see Fig. S5).

10.1128/mSphereDirect.00153-19.5 FIG S5 Phase-contrast images of DAB-labeled *S. salmonicida* (A to G) and *G. intestinalis* (H to K) transfectants. Transfectants expressing APEX were spotted on a poly-lysine-coated microscopy slide, fixed with 2% glutaraldehyde in 100 mM cacodylate buffer with 2 mM CaCl_2_, and treated with 0.5 mg/ml DAB and 300 µM H_2_O_2_ for 15 min. Cells were washed with cacodylate buffer and mounted with VectaShield before imaging with phase-contrast microscopy. Cells with white or black deposits are positive for APEX-catalyzed staining. Scale bars = 2 µm. Download FIG S5, PDF file, 0.2 MB. Copyright © 2019 Ástvaldsson et al. 2019 Ástvaldsson et al. This content is distributed under the terms of the Creative Commons Attribution 4.0 International license.

**FIG 4:**
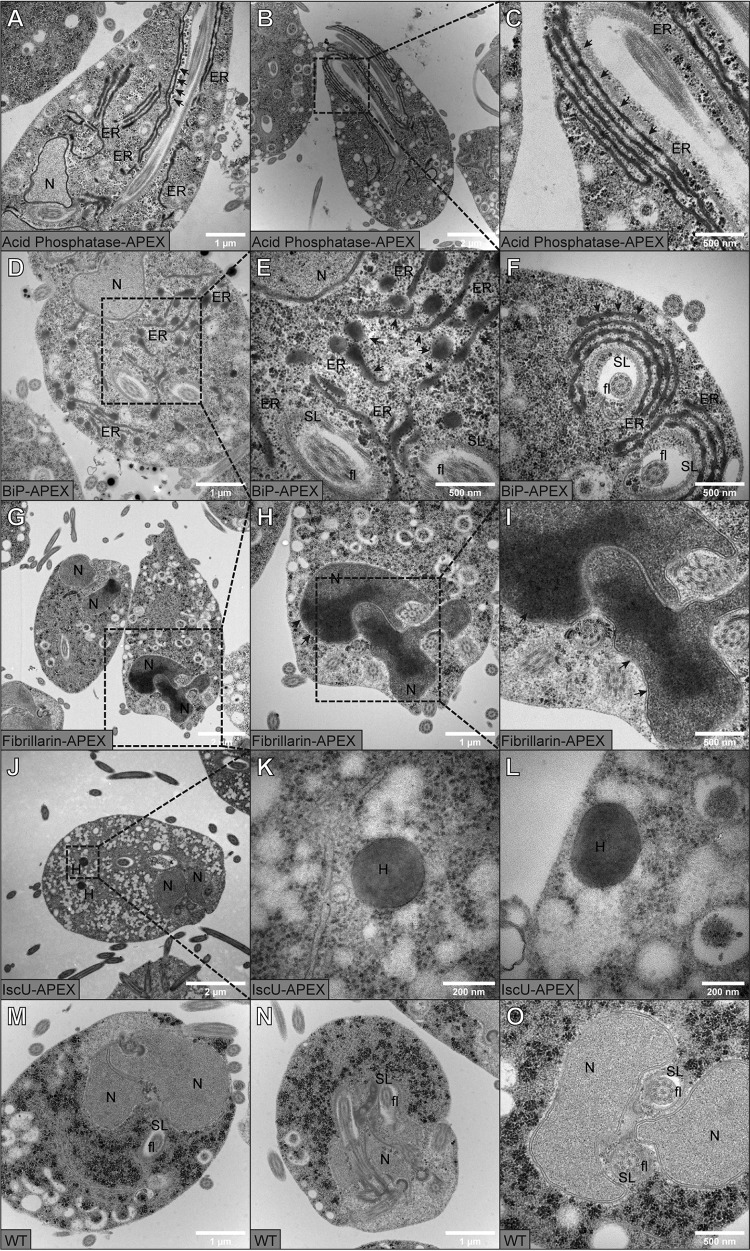
Transmission electron microscopy (TEM) images of DAB-stained *S. salmonicida* transfectants and WT. (A to C) Acid phosphatase-APEX; (D to F) BiP-APEX; (G to I) fibrillarin-APEX; (J to L) IscU-APEX; (M to O) WT. Cultures were grown in LYI medium containing 100 µM hemin. Cells are fixed with 2% glutaraldehyde in 100 mM cacodylate buffer with 2 mM CaCl_2_ and treated with 0.5 mg/ml DAB and 200 µM H_2_O_2_ for 15 min. Samples were postfixed in 2% osmium tetroxide in 0.1 M phosphate buffer following dehydration in ethanol and acetone. Samples were embedded in LX-112 resin, and 50- to 60-nm sections were cut. Samples were viewed at 80 kV on a Hitachi HT 7700 lens and imaged with a Veleta camera. APEX-catalyzed DAB deposition appears as higher-contrast areas at the site of the tagged protein. Some areas with labeling are indicated by arrows. N, nucleus; SL, striated lamina; fl, flagella; H, hydrogenosome; ER, endoplasmic reticulum. Scale bars are 2 µm in panels B, G, and J; 1 µm in A, D, H, M, and N; 500 nm in C, E, F, I, and O; and 200 nm in K and L.

In determination of the localizations of S. salmonicida ER-localized fusion proteins, acid phosphatase and BiP, display prominent labeling in TEM micrographs. The perinuclear area showed a clear signal, and the nuclear-posterior area displayed an interconnected ER network. The ER label was found to track as layered sheets along the recurrent flagellar axes outside the striated lamina that enclose the recurrent flagella. In cross-sections of tapering cell ends, these sheets were bridged in an apparent S shape. The ER sheets tapered but were always present toward the cell posterior where the recurrent flagella exit the cell body (Fig. 4A to F). The labeled regions are consistent with the distribution of ER in S. salmonicida, as described by Jørgensen and Sterud (20).

Labeling of the fibrillarin-APEX fusion strain revealed darkened nuclei, with the anterior ends showing a particularly intense stain. In some cells, there were lobes of intense stain in other parts of the nucleus. These were most of the time seen as connected at the anterior ends. The organization of the nucleolus in S. salmonicida appears to be reminiscent of the diffuse nucleolar organizing regions seen in Giardia trophozoites (21), although they appear to occupy a proportionally larger volume of the nucleus. We did not observe any distinct granular nucleoli similar to those recently demonstrated in Giardia spp. (Fig. 4G to I) (22).

The IFT46 protein fusion is detected as a general darkening of the flagella with highly localized foci on the flagella, expected to be IFT particles. Even though we were able to easily visualize the DAB stain by light microscopy, we were unable to detect any labeled structures by TEM. We attribute this failure to be due to the comparative rarity and minute size of these stained cellular features.

IscU is clearly detectable as intensely staining dark vesicles that are bounded by double membranes. In some micrographs, it was possible to see intensely stained granulated areas in the matrix. The organelle sizes and their cellular distribution are fully consistent with previous immuno-EM and TEM data for S. salmonicida hydrogenosomes (Fig. 4J to L) (4).

SS50377_12178 shows a clear perinuclear labeling in DAB-stained whole cells (Fig. S4F). Surprisingly, we were unable to demonstrate any convincing DAB-derived deposits in the nuclear area by TEM.

TEM micrographs of the SS50377_10316 APEX fusion showed intense staining of the nucleus-abutting section of the basal body pockets. In dividing cells with duplicated cellular structures, the labeled basal body pockets were found to be paired up before nuclear division (Fig. 3C and D). Future studies attempting to understand cell division dynamics in S. salmonicida would be able to use SS50377_10316 as a marker to track basal body nuclear dynamics.

**FIG 5:**
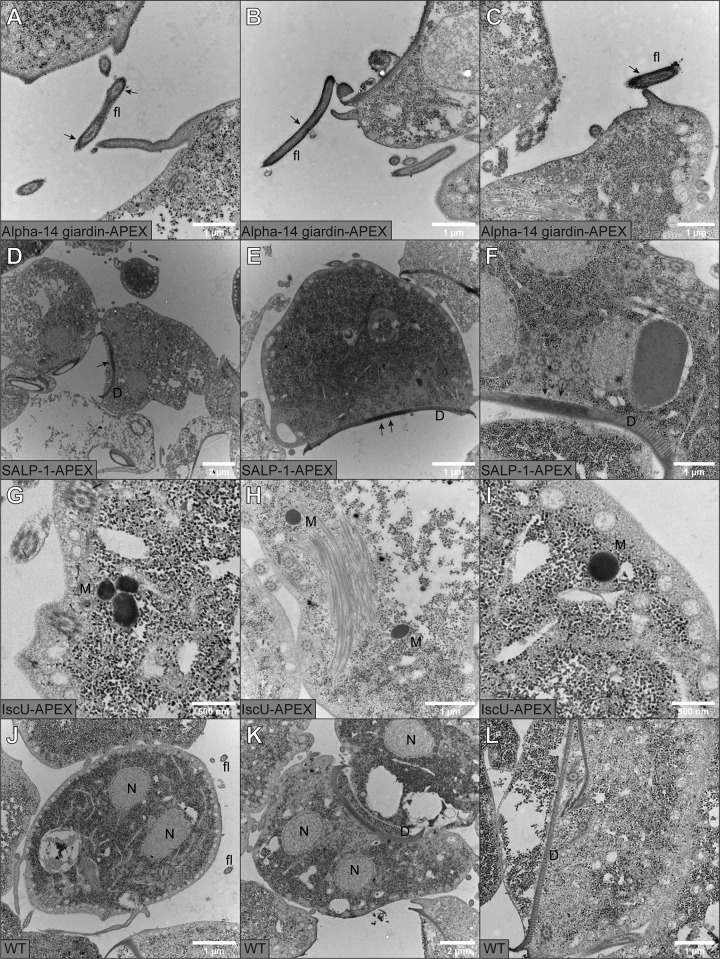
Transmission electron microscopy (TEM) images of DAB-stained *G. intestinalis* transfectants and WT. (A to C) Alpha 14-giardin-APEX; (D to E) SALP-1-APEX; (G to I) IscU-APEX; (J to L) WT. Cultures were grown in TYDK medium supplemented with 100 µM hemin. The cells were fixed with 2% glutaraldehyde in 100 mM cacodylate buffer with 2 mM CaCl_2_ and labeled with 0.5 mg/ml DAB and 300 µM H_2_O_2_ for 15 min. Samples were postfixed in 2% osmium tetroxide in 0.1 M phosphate buffer following dehydration in ethanol and acetone. Samples were embedded in LX-112 resin, and 50- to 60-nm sections were cut. Samples were viewed at 80 kV on a Hitachi HT 7700 lens and imaged with a Veleta camera. Scale bars are 2 µm in panels D and K; 1 µm in A, B, C, E, F, H, J, and L; and 500 nm in G and I.

DAB staining of G. intestinalis alpha-14 giardin transfectants results in very prominently labeled flagella. TEM sections revealed that the DAB stain was primarily associated with the flagellar plasma membrane (Fig. 5A to C). A lower intensity of staining was also present in the plasma membrane. This labeling is consistent with that seen by alpha-14 giardin antisera (19).

Whole cells with SALP-1 labeling displayed dark outlines of the ventral disc. TEM imaging of the cells showed a clear ventral disc labeling which increased at the disc periphery. At higher magnification, the SALP-1 signal is found to be associated with the ventral disc microribbons, as previously postulated (Fig. 5D to F) (17).

The Giardia IscU signal was seen in membrane-bounded vesicles distributed in the cytosol (Fig. 5G to I). This localization is fully consistent with mitosome localization in Giardia intestinalis (5).

## DISCUSSION

In this study, we describe the successful optimization of a proximity labeling system based on the APEX in the diplomonad S. salmonicida. The method was also showed to be applicable to G. intestinalis. The flexibility of APEX allowed us to use both fluorescent- and osmiophilic-yielding substrates with the same transfectant strains. We further expect that the APEX toolkit is expandable by utilization of the well-developed commercial substrate catalog available for HRP. As an example, recent work has seen the development of spatially resolved *in vivo* proximity labeling using APEX in combination with the novel substrate biotin-phenol (23).

Robust APEX activity in both diplomonads required the addition of exogenous hemin to the growth media. This likely reflects the low abundance of the heme cofactor, which is needed for APEX activity, in the basal growth media of the two diplomonads. We determined that the optimal conditions to load the cells with heme for APEX activation were well below toxicity levels of the cells and that signal could be further modulated by H_2_O_2_ titration. One substantial benefit to APEX is the lack of disulfides and calcium-binding sites which render it more versatile as a fusion partner than HRP. In line with this, we have been able to demonstrate that APEX is active in a broad selection of cellular compartments, including the ER, nucleus, plasma membrane, the perinuclear region, cytoskeleton, and the MROs. The observed signal strength for some constructs, especially in TEM, was sometimes weak and difficult to find. For example, even though S. salmonicida IFT46 and SS50377_12178 yielded robust signal using Amplex UltraRed and in whole DAB-stained cells, we were unable to determine the corresponding labeled regions in TEM. For IFT46, we believe that the signal is spatially confined to highly specific regions, presumed to be complex B of IFT particles (24), so we did not chance a productive cross-section. There are flagellar isolation protocols developed for use in Giardia spp. (25) which if adapted to Spironucleus spp. would increase the chances of observing stained IFT particles.

Recently, a further evolved version of ascorbate peroxidase called APEX2 was shown to offer labeling with increased sensitivity and robustness for weakly expressed proteins (8, 26). This protein was previously successfully employed by Zumthor et al. to demonstrate the ultrastructural localization of proteins in G. intestinalis (8). We have performed preliminary experiments using APEX2 derived from soybean in our diplomonad systems using the conditions we optimized for APEX in the present study. We observed that APEX2 fusions display increased activity but also yielded increased background staining (see Fig. S6). Based on this, we recommend the use of APEX rather than APEX2 in Spironucleus salmonicida, at least until reaction conditions can be optimized. The previous demonstration of APEX2 function in G. intestinalis (8) indicates that the use of either APEX or APEX2 should be considered depending on the expression level or cellular location of the protein under study. This could be relevant to compensate for the apparent lower labeling efficiency we observed for non-membrane-bounded proteins in Giardia spp.

10.1128/mSphereDirect.00153-19.6 FIG S6 Comparison between APEX and APEX2. Two proteins, annexin 5 and IscU, were tagged with both APEX-V5 and APEX2-V5 to compare the activities of the peroxidases. *S. salmonicida* transfectants stably expressing the constructs were washed with HBSS-G and spotted on a poly-lysine*-*coated microscopy slide. (A, C, E, and G) DAB*-*treated cells were fixed with 2% glutaraldehyde in 100 mM cacodylate buffer with 2 mM CaCl_2_ and reacted with 0.5 mg/ml DAB substrate and 300 µM H_2_O_2_ for 15 min. Cells washed with cacodylate buffer and PBS and then mounted in VectaShield. Cells with white or black deposits are positive for APEX-catalyzed staining. (B, D, F, and H) Amplex UltraRed*-*treated cells were fixed with 2% paraformaldehyde in PBS and reacted with 50 µM Amplex UltraRed and 200 µM H_2_O_2_ for 30 min. The cells were then washed with PBS and mounted with VectaShield with DAPI. Cells with red deposits in the anterior of the cells are positive for APEX or APEX2 staining. Scale bars = 10 µm. Download FIG S6, PDF file, 0.4 MB. Copyright © 2019 Ástvaldsson et al. 2019 Ástvaldsson et al. This content is distributed under the terms of the Creative Commons Attribution 4.0 International license.

It is clear from our data that using the Amplex UltraRed substrate in combination with APEX fusions yield reliable localization information. However, we were clearly able to achieve higher localization resolution by antibody-based labeling using the V5 tag than using the Amplex UltraRed substrate. We attribute this to the tendency of resorufin to leach from its immediate site of generation. Thus, presently, the best combination for simultaneous high-resolution localization using fluorescence microscopy and TEM would be offered by an APEX-epitope tag combination.

Our approach was able to confirm the ultrastructural localization of 9 diplomonad proteins, including a protein in S. salmonicida with previously unclear or unknown localization. As might be expected from a larger protein tag, we had some issues making fusions to some of our target proteins. For a few proteins, we were unable to recover transfected cells despite several attempts, indicating issues with toxicity of the fusion protein. Several additional proteins showed no expression or a wrongly sized polypeptide in the recovered transfectants. However, we did not observe any evidence of tag-induced mistargeting, and we believe that most of the toxicity issues are not specifically connected to APEX and can be resolved by switching the tagging termini, by integration on the chromosome, or by using an inducible expression system. The difficulties in making fusions were especially pronounced in Giardia intestinalis, which agree with our previous experiences that protein tagging is more likely to be more successful in S. salmonicida than in Giardia intestinalis. In this sense, S. salmonicida can be utilized as a viable comparative model for localization in case the protein is refractory to localization in Giardia intestinalis and an ortholog exists in S. salmonicida.

We believe that the APEX proximity labeling system will be of great use in the study of the intricate cell biology and cytoskeletal systems present in diplomonad cells and many other protists with transfection possibilities. Our anticipation is that future developments to the APEX system might be able to extend its use in diplomonads to include spatially resolved enzymatic tagging and enable compartment-resolved proteomics.

## MATERIALS AND METHODS

### Cell culture.

Cultures were grown in axenic cultures in tightly closed slanted polypropylene tubes (Nunc). S. salmonicida (ATCC 50377), obtained from Atlantic salmon, was grown in modified LYI medium (12) at 16°C, while G. intestinalis WB clone 6 (ATCC 50803) was grown in TYDK (Diamond's TYI-S-33 supplemented with bile according to the methods of Keister) culture medium at 37°C (27).

### Vector constructions.

The pea APEX, APX^W41F^, and the soybean APEX2 genes were PCR amplified from the pUC57 Apex plasmid (Addgene identifier [ID] 40306), the pCAG APX W41F plasmid (Addgene ID 40307), and the pcDNA3 Connexin43-GFP-APEX2 plasmid (Addgene ID 49385), respectively, using corresponding primers. The C-terminal primers contained overhangs of either the V5 or the 3× hemagglutinin (3×HA) epitope tags to generate fusions (see Table S1). The products were inserted into the pSpiro-PAC (12) and pPAC (28) vectors using restriction digestion for transfections in S. salmonicida and G. intestinalis, respectively. The sizes of the inserts are as follows: APX^W41F^-3×HA and APEX-3×HA, 828 bp and 30.28 kDa, respectively; APEX-V5 and APEX2-V5, 789 bp and 28.3 kDa, respectively. Coding sequences of target genes were amplified from genomic DNA using PCR and included a 100- to 400-bp upstream putative promoter region. The primer sequences can be found in Table S1. Primer sequences used for amplification of the S. salmonicida genes NADH, SS50377_10316, IscU, IscS, SHMT, and PFOR5 can be found in reference 4; those for IFT46, BiP, and fibrillarin can be found in reference 12, and those for annexin 5 can also be found in reference 12. Restriction digestion was used to insert the genes into the expression vectors. All constructs were verified using Sanger sequencing at SciLifeLab, Uppsala, Sweden. Transfection of S. salmonicida was done according to reference 12, while G. intestinalis was transfected as described in reference 4. Transfectants were selected using 50 µg/ml puromycin and grown under constant selective pressure.

10.1128/mSphereDirect.00153-19.7 TABLE S1 List of cloning primers and oligonucleotide sequences. Download Table S1, PDF file, 0.10 MB. Copyright © 2019 Ástvaldsson et al. 2019 Ástvaldsson et al. This content is distributed under the terms of the Creative Commons Attribution 4.0 International license.

### Immunofluorescence assay.

Preparations of S. salmonicida and G. intestinalis transfectants for immunofluorescence assay mostly followed the guidelines in reference 4. Cultures were pelleted by centrifugation and washed with Hanks balanced salt solution with glucose (HBSS-G) (S. salmonicida) or phosphate-buffered saline (PBS) (G. intestinalis). Cells were spotted on poly-lysine-coated microscope slides (Thermo Fisher Scientific catalog no. ER-208B-CE24) and fixed with 2% paraformaldehyde (Sigma-Aldrich catalog no. 344198) in PBS for 20 min at 37°C. Fixative was quenched with 0.1 M glycine for 10 min at room temperature (RT). Cells were permeabilized with 0.2% Triton X-100 in PBS for 20 min at RT and blocked with 2% bovine serum albumin (BSA) in 0.5% Triton X-100 in PBS for 1 h at RT or overnight (O/N) at 4°C. V5 epitope-tagged proteins were detected using anti-V5 monoclonal antibody SV5-Pk1 and diluted 1:750 (Abcam catalog no. AB27671) in block solution at RT for 1 to 2 h. 3×HA-tagged proteins were detected using either an Alexa Fluor 488-conjugated anti-HA monoclonal antibody HA.11, diluted 1:250 (Nordic BioSite catalog no. 901509) in block solution or primary rabbit anti-HA monoclonal antibody C29F4, and diluted 1:1,600 (Cell Signaling catalog no. 3724S) in block solution at RT for 1 to 2 h. Primary antibodies were detected using Alexa Fluor 488-conjugated goat anti-mouse polyclonal antibody, diluted 1:800 (Life Technologies catalog no. A11029) in block solution at RT for 1 h or Alexa Fluor 488-conjugated goat anti-rabbit polyclonal antibody, and diluted 1:350 (Life Technologies catalog no. A11008) in block solution at RT for 1 h. Slides were mounted using VectaShield containing 4′,6-diamidino-2-phenylindole (DAPI; Vector Laboratories catalog no. H-1200). Cells were viewed using a Zeiss Axioplan 2 fluorescence microscope (Carl Zeiss GmbH), and images were processed using Zen lite 2012 (blue edition) version 1.1.2.0, ImageJ-Fiji version 1.51d, and Adobe Illustrator CC.

### Western blotting.

Preparations of cell lysates for expression analyses essentially followed the guidelines in reference 28. Cultures were pelleted by centrifugation and washed with HBSS-G (S. salmonicida) or PBS (G. intestinalis). Cells were normalized to 0.05 optical density (OD) units/10 µl at 600 nm in a spectrophotometer. Samples were boiled for 10 min in 1× Laemmli buffer with 100 mM dithiothreitol (DTT). Protein separations was performed using precast polyacrylamide gels (Bio-Rad Mini-Protean Any-kD TGX stain free, catalog no. 456-8125) and transferred to polyvinylidene difluoride (PVDF) (Bio-Rad catalog no. 162-0177). Membranes were blocked with 5% dry milk (Semper) in 0.05% Tween 20 (Sigma-Aldrich catalog no. P9416) in Tris-buffered saline (TBS) for 1 h. Proteins were detected with a primary mouse anti-V5 monoclonal antibody, SV5-Pk1 (Abcam catalog no. AB27671), and diluted 1:2,000 incubated at RT for 2 h and a secondary HRP-conjugated rabbit anti-mouse polyclonal antibody (Dako catalog no. P0161) diluted 1:10,000 and incubated at RT for 1 h. Developing was done using Clarity Western ECL substrate (Bio-Rad catalog no. 170-5061), and blots were imaged using the Bio-Rad ChemiDoc MP+ system. Images were processed using Bio-Rad Image Lab version 4.0 and Adobe Illustrator CC.

### Amplex UltraRed assay.

Cultures were grown in culture medium supplemented with 100 mM hemin (Sigma-Aldrich catalog no. H9039) if not stated otherwise. Cultures were pelleted and washed with HBSS-G (S. salmonicida) or PBS (G. intestinalis) and fixed with 2% paraformaldehyde in PBS for 20 min at 37°C. Fixative was quenched with 0.1 M glycine for 10 min at room temperature. If cells were doubly stained with antibodies, the cells were permeabilized and blocked as for the immunofluorescence assay. Cells were labeled for 30 min on ice with labeling solution that contained 50 µM Amplex UltraRed substrate (Thermo Fisher Scientific catalog no. A36006) and 200 µM H_2_O_2_ (Sigma-Aldrich catalog no. 216763) if not stated otherwise. Cells were mounted, viewed, imaged, and processed the same way as the immunofluorescence assay images.

### SIM.

Cells were prepared the same way as for the Amplex UltraRed assay, except prior to mounting of the slides with Vectashield without counterstain (Vector Laboratories catalog no. H-1000), the cells were stained with 2 µg/ml DAPI (Sigma-Aldrich catalog no. 32670) for 10 min at room temperature. Cells were viewed using a Zeiss LSM710 with Zeiss Elyra S.1 structured illumination microscope (SIM) (Carl Zeiss GmbH) for superresolution. Images were processed the same way as the immunofluorescence assay images.

### DAB proximity labeling on slides.

Cultures were grown and washed as for the Amplex UltraRed assay. The cells were resuspended in assay buffer (100 mM cacodylate [Sigma catalog no.C-0250], 2 mM CaCl_2_ [pH 7.4]) and spotted on a poly-lysine-coated slide (Thermo Fisher Scientific catalog no. ER-208B-CE24). Cells were fixed with 2% glutaraldehyde (Sigma-Aldrich catalog no. G5882) in assay buffer for 60 min, washed with assay buffer, and quenched with 20 mM glycine in assay buffer for 5 min. After washing, the cells were labeled for 15 min with 3,3′-diaminobenzidine (DAB) labeling solution containing 0.5 mg/ml (1.4 mM) DAB (Sigma-Aldrich catalog no. D5905) and 300 µM H_2_O_2_ in assay buffer, unless stated otherwise. Slides were mounted using VectaShield without DAPI and processed the same way as the immunofluorescence samples.

### DAB proximity labeling for TEM.

Bulk samples for TEM were prepared using the same procedure as on slides. Pellets are fixed in 2% glutaraldehyde in assay buffer with 2 mM CaCl_2_ for 60 min at 4°C with rotation. Cells were pelleted by centrifugation and washed 2× with assay buffer before fixative quenching with 20 mM glycine in assay buffer for 5 min at 4°C with rotation. Cells were washed the same way as before and labeled with the 0.5 mg/ml DAB labeling solution and 300 µM H_2_O_2_, if not stated otherwise, for 20 min at 4°C with rotation. After extensive washing with assay buffer, 20 µl of cell suspension was spotted on a poly-lysine-coated microscope slide, incubated for 20 min at 4°C to allow for attachment, washed 4× with PBS to remove assay buffer, and mounted with VectaShield. Viewing and processing of images were done the same way as for the immunofluorescence assay. The remaining pellet was pelleted once more before resuspending in 2% glutaraldehyde and 1% formaldehyde in assay buffer.

### TEM.

Processing and viewing were done similar to what was performed in reference 12. Briefly, pellets were postfixed in 2% osmium tetroxide in 0.1 M phosphate buffer for 2 h at 4°C following dehydration in ethanol and acetone before samples were embedded in LX-112 resin (Ladd, Burlington, VT). Sections of 50 to 60 nm were cut using an Ultracut UCT ultramicrotome (Leica, Vienna, Austria), examined at 80 kV using an HT 7700 lens (Hitachi, Tokyo, Japan), and imaged with a Veleta camera (Olympus Soft Imaging Solutions GmbH, Münster, Germany).
