# Supplementary figures and images for: Proximity Staining Using Enzymatic Protein Tagging in Diplomonads
Source: mSphere. 2019 Mar 20;4(2):e00153-19. doi: 10.1128/mSphereDirect.00153-19 (PMC6429047; doi:10.1128/mSphereDirect.00153-19)

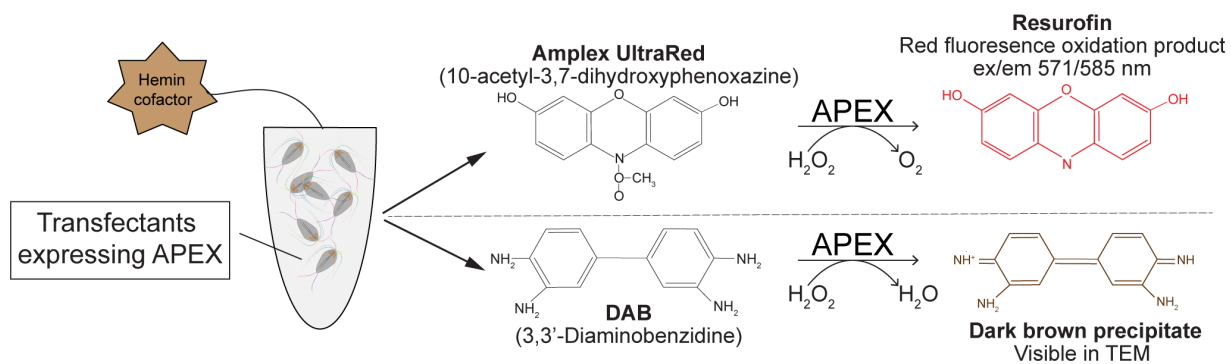

Supplement: FIG S1 [file mSphereDirect.00153-19-sf001.pdf]

**A.**

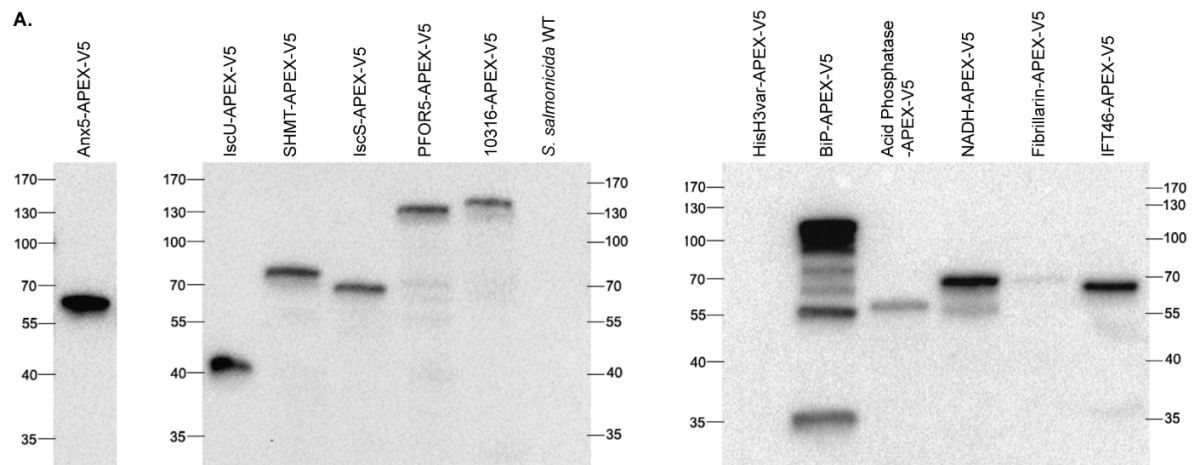

**B.**

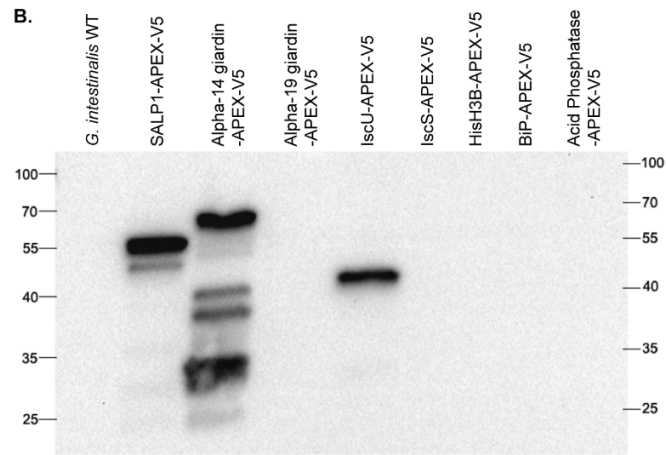

Supplement: FIG S2 [file mSphereDirect.00153-19-sf002.pdf]

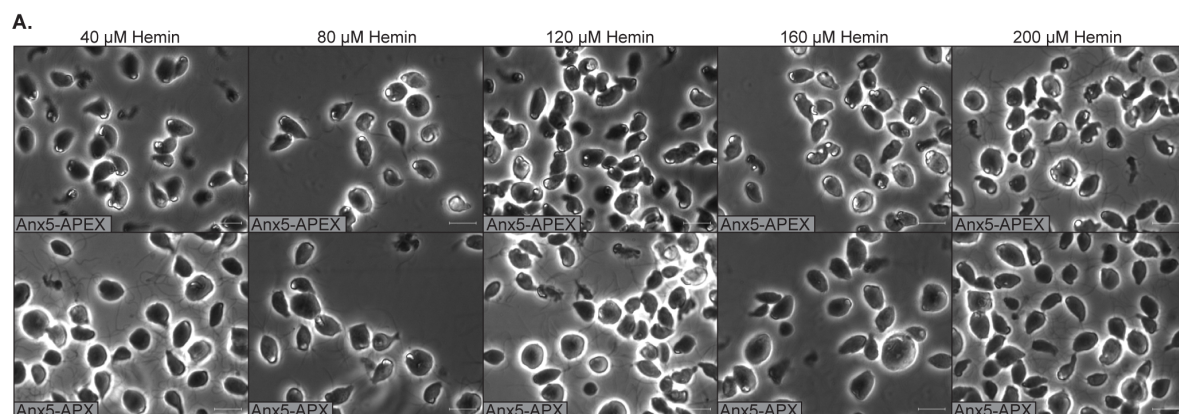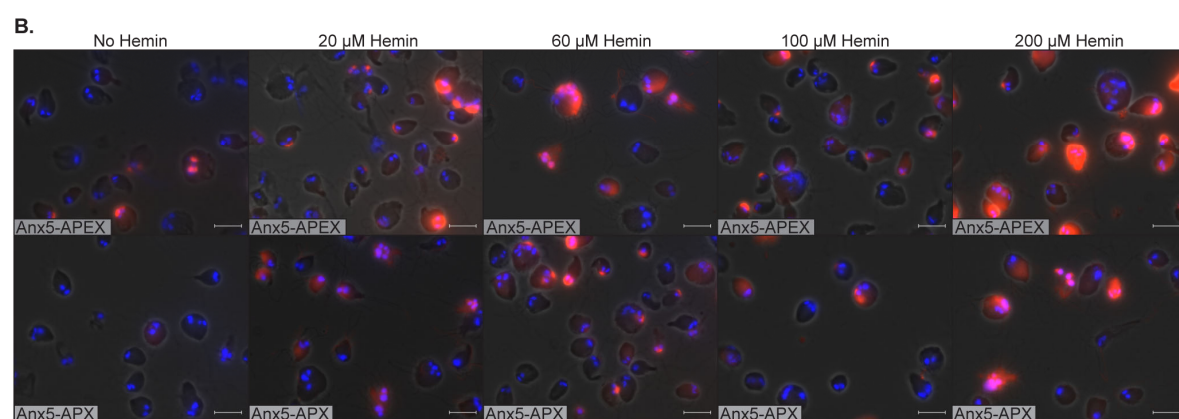

Supplement: FIG S3 [file mSphereDirect.00153-19-sf003.pdf]

A.

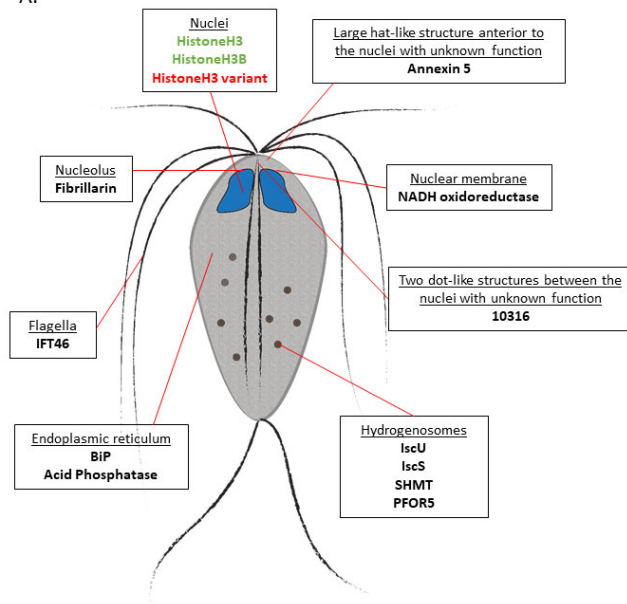

B.

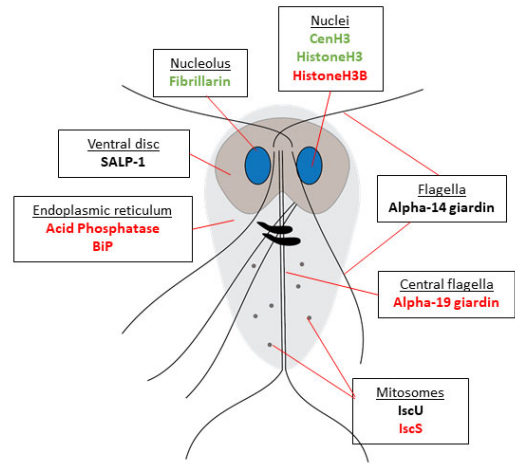

Supplement: FIG S4 [file mSphereDirect.00153-19-sf004.pdf]

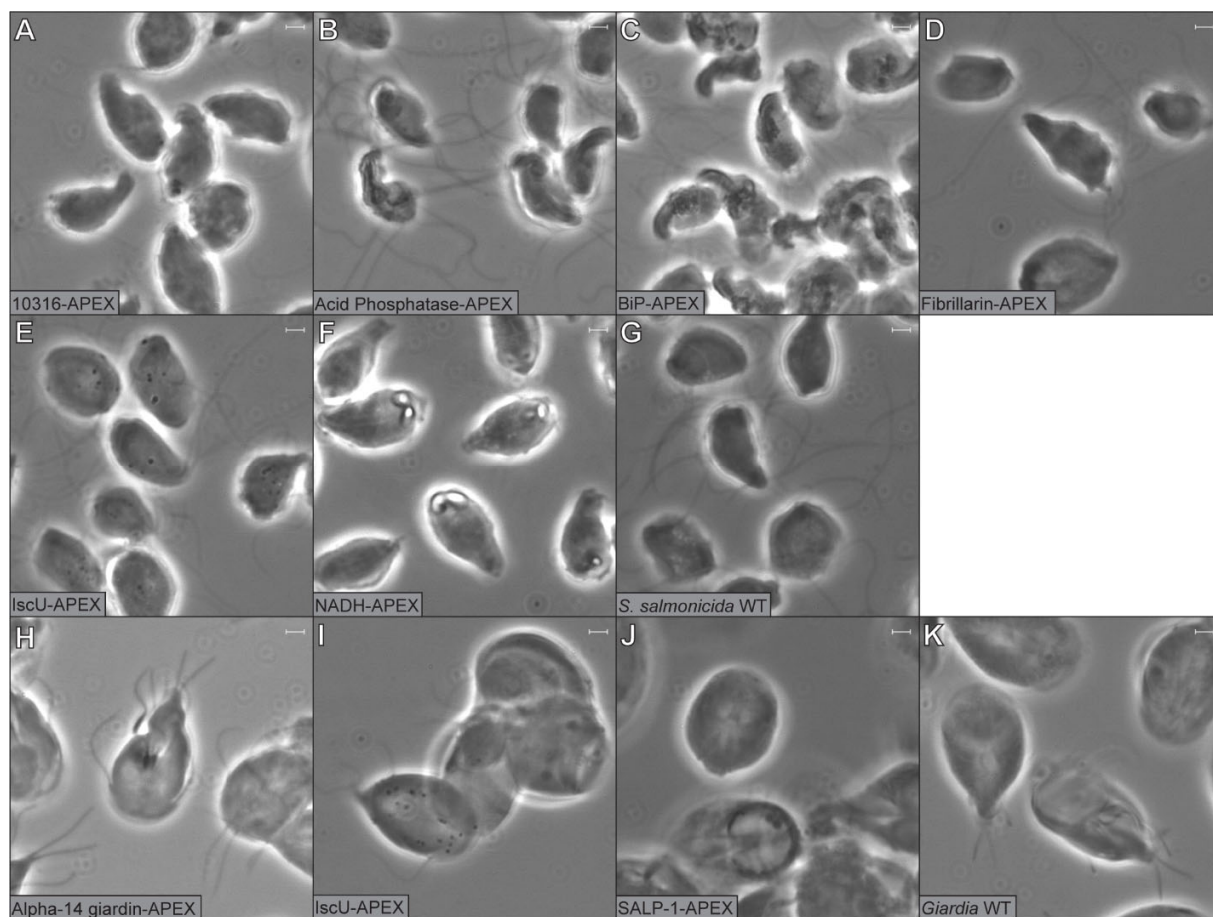

Supplement: FIG S5 [file mSphereDirect.00153-19-sf005.pdf]

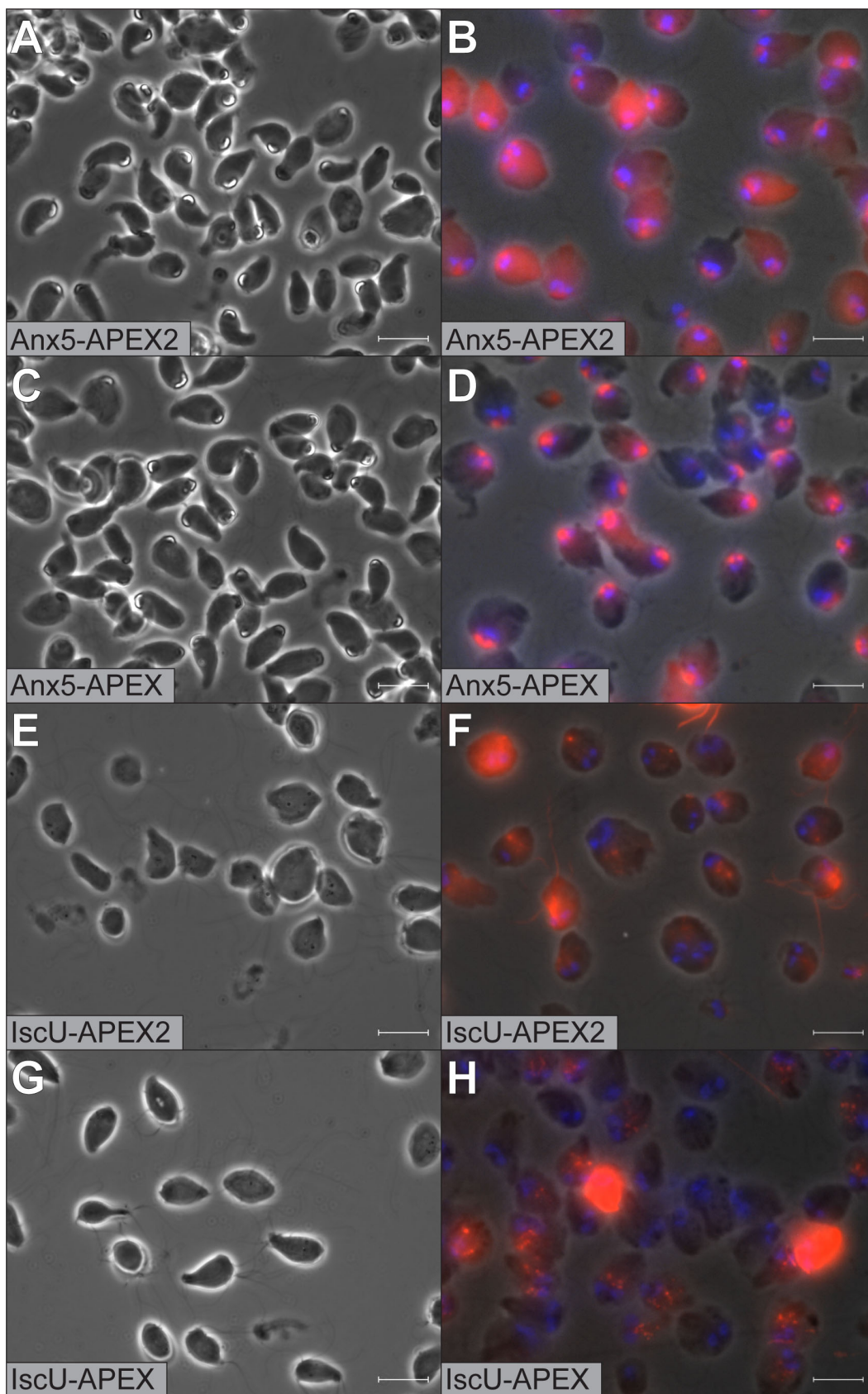

Supplement: FIG S6 [file mSphereDirect.00153-19-sf006.pdf]
