# Supplementary material for: Proximity Staining Using Enzymatic Protein Tagging in Diplomonads
Source: mSphere. 2019 Mar 20;4(2):e00153-19. doi: 10.1128/mSphereDirect.00153-19 (PMC6429047; doi:10.1128/mSphereDirect.00153-19)
Supplement: TABLE S1 [file mSphereDirect.00153-19-st001.pdf]

Name	Oligonucleotide sequence
tdAPX-NotI-F	TATGCGGCCGCCCGGCGGATCAGGCTCTATGGGCAAGAGCTATCCCACC
tdAPX-HA-ApaI-R	TATGGGCCCGGCGTAGTCAGGGACATCATAGGGGTAGGCTTCAGCAAATCCAAGCTCAG
C-APEX-NotI-F	TATGCGGCCGCCCGGCGGATCAGGCTCTGGAAAGTCATACCCAACCGTGAGC
C-APEX-3xHA-ApaI-R	TATGGGCCCGGCGTAGTCAGGGACATCATAGGGGTAAGCATAGTCAGGAACATCGTACGGATATGCGTAATCTGGCACGTCGTAGGGATATGCCTCGGCGAATCCCAGTTCAG
APEX-NotI-F	GCGGCCGCccGGCGGATCAGGCTCTGG
APEX-V5-ApaIR	GGGCCCCGTAGAATCGAGACCGAGGAGAGGGTTAGGGATAGGCTTACCTGCCTCGGCGAATCCCAGTTC
APEX2-NotI-F	ATAGCGGCCGCCCGGCGGATCAGGCTCTGGAAAGTCTTACCCAACTGTGAGTGCTGATTACCAGGAC
APEX2-V5-ApaI-R	TATGGGCCCCGTAGAATCGAGACCGAGGAGAGGGTTAGGGATAGGCTTACCGGCATCAGCAAACCCAAGCTCG
Spiro.HistoneH3-F	ATATACGCGTTACAAGGTATGATAGGAGCCGTCTGGAGT
Spiro.HistoneH3-R	TATGCGGCCGCCACTTCTTGTGGTCAGGCTGGG
Spiro.HistoneH3B-F	ATATACGCGTATCCACTTCAGCAACGAGGATAAATGTATG
Spiro.HistoneH3B-R	TATGCGGCCGCCACTTCATGGAAAGAGCCCATGC
Spiro.HistoneH3var-F	ATATACGCGTATGAATAGAGAATATGTAAGGAATATAGCGAAGGTTTAACTG
Spiro.HistoneH3var-R	TATGCGGCCGCCATTAAAAATTCCATGATGGTCTTAGAATCATTATAAGC
Spiro.AcidPhostphatase-F	ATATTTAATTAAGGTTGATAACACAATCTTATCAGCAGATCGAT
Spiro.AcidPhostphatase-R	TATGCGGCCGCCACTGCGCGAACTCCGTTTC
Giardia.IscU-F	ATATACGCGTCAAGCTTGTAGGCCATTGGCTCTAT
Giardia.a14-giardin-F	TATACGCGTGCTCCAGACCATAGGGGGCACCAGGG
Giardia.a14-giardin-R	TATGCGGCCGCAACTCGAGAGTCATCGGCCTGTGCTG
Giardia.a19-giardin-F	TATACGCGTGGTGGCACGAACACCTTTAGAAACAGTATGGCTTGTGG
Giardia.a19-giardin-R	TATGCGGCCGCCAGTCGCCGCGGGGAGTCGAGGATTCGC
Giardia.IscU-R	TATGCGGCCGCCAAGAAGACTTTGATACCTGTATCTTGCTTCC
Giardia.IscS-F	ATATACGCGTGGTAACATCGTTAAGAACACGAGCTCAC
Giardia.IscS-R	TATGCGGCCGCTAGTCATGCTTCCACTCTATGCTCTTTGTATC
Giardia.HistoneH3-F	ATATACGCGTCTGCGATCTCATACGCATGGAGAA
Giardia.HistoneH3-R	TATGCGGCCGCTACTTGCCCTTCCTGTACTCGGGC
Giardia.HistoneH3B-F	ATATACGCGTTATCCGTTGCTGATCGACAGGAAG
Giardia.HistoneH3B-R	TATGCGGCCGCTACAGCATACTTCCATGAATGCCGTT
Giardia.CenH3-F	ATATACGCGTTGACTGTGCTGACTCGATTCACTGA
Giardia.CenH3-R	TATGCGGCCGCTACCGTAGTGAATTTAAGTTGCGCTGC
Giardia.Fibrillarin-F	ATATACGCGTGGCTGCCGTTATAAGAACGTCTCCA
Giarda.Fibrillarin-R	TATGCGGCCGCCACGCTGCCTTGACACGGA
Giardia.BiP-F	ATATACGCGTTGATAGCACAGACAATGTGCTGAGTTAGA
Giardia.BiP-R	TATGCGGCCGCTATGCGGCCGCTAGAGTTCATCTTTTTCTGCATAGTCGTACTCAG
Giardia.AcidPhosphatase-F	ATATTTAATTAAAGGATTCAGTCAGCCGTTCGCTATG
Giardia.AcidPhosphatase-R	TATGCGGCCGCCAGTTCGCTTTGATCTCCTGAGCG
Giardia.SALP-1-F	TATACGCGTCCACTTAATGAGCAAGTTGGAATAAAACCG
Giardia.SALP-1-R	TATGCGGCCGCAGGACACCCACTCCCGTGTAGATATTACG
